# Temperature Drift Compensation for Deep-Well Drill Bit State Parameters Using Electric Eel Foraging Optimization and Physics-Informed Extreme Learning Machine

**DOI:** 10.3390/s26216930

**Published:** 2026-11-02

**Authors:** Jiawen Xu, Zhenhuan Yi, Zhenbao Li, Yongyong Li, Hengshuo Zhang, Yong Zhu

**Affiliations:** 1 National Research Center of Pumps, Jiangsu University, Zhenjiang 212013, China; 2222511095@stmail.ujs.edu.cn (J.X.); 2212411027@stmail.ujs.edu.cn (H.Z.); 2 College of Petroleum, China University of Petroleum-Beijing at Karamay, Karamay 834000, China; y2024216880@st.cupk.edu.cn; 3 Institute of Engineering Technology, PetroChina Western Drilling Engineering Co., Ltd., Karamay 834000, China; lzbgcy@cnpc.com.cn (Z.L.); leey3515@cnpc.com.cn (Y.L.)

**Keywords:** deep well drilling, measurement while drilling, temperature drift compensation, physics-informed extreme learning machine, electric eel foraging optimization algorithm

## Abstract

In deep and ultra-deep drilling, high downhole temperatures cause appreciable thermal drift in the strain-gauge bridge of the bit parameter recorder, degrading the measurement fidelity of weight-on-bit (WOB) and torque. This paper presents a temperature-drift compensation scheme combining electric eel foraging optimization (EEFO) with a physics-informed extreme learning machine (PIELM). Four physical priors are formulated as constraints: trend consistency and regression deviation are embedded into the output-weight solution through an augmented linear system, while proportional relationship and continuity enter the fitness function as penalty terms. EEFO performs the hyperparameter search; enhancement strategies addressing its population-diversity decay and limited final-stage accuracy are examined under an equal function-evaluation budget, and the deployed configuration is determined by the ablation evidence. Under leakage-free nested cross-validation, hyperparameter optimization reduces the WOB RMSE from 0.712 kN with fixed hyperparameters to 0.246 kN, a 2.9-fold improvement, while a ridge-regularized ELM baseline reaches 0.708 kN, indicating that the gain originates from the hyperparameter optimization itself. Equal-budget ablation on 13 classical benchmark functions ranks the dynamic-boundary quasi-opposition-based learning variant as the most effective single strategy, and the deployed configuration attains the lowest mean fitness on the target problem. Downhole evaluation is formulated as a consistency-verification framework, with no downhole load ground truth and the WOB voltage lying outside the training range. The apparent drift elimination under in-sample mapping does not survive leakage-free mapping protocols (the WOB elimination rates are 0.3%, 0.0%, and 5.1% under P2–P4) and is therefore not interpreted as a compensation-accuracy figure.

## 1. Introduction

**Figure 1:**
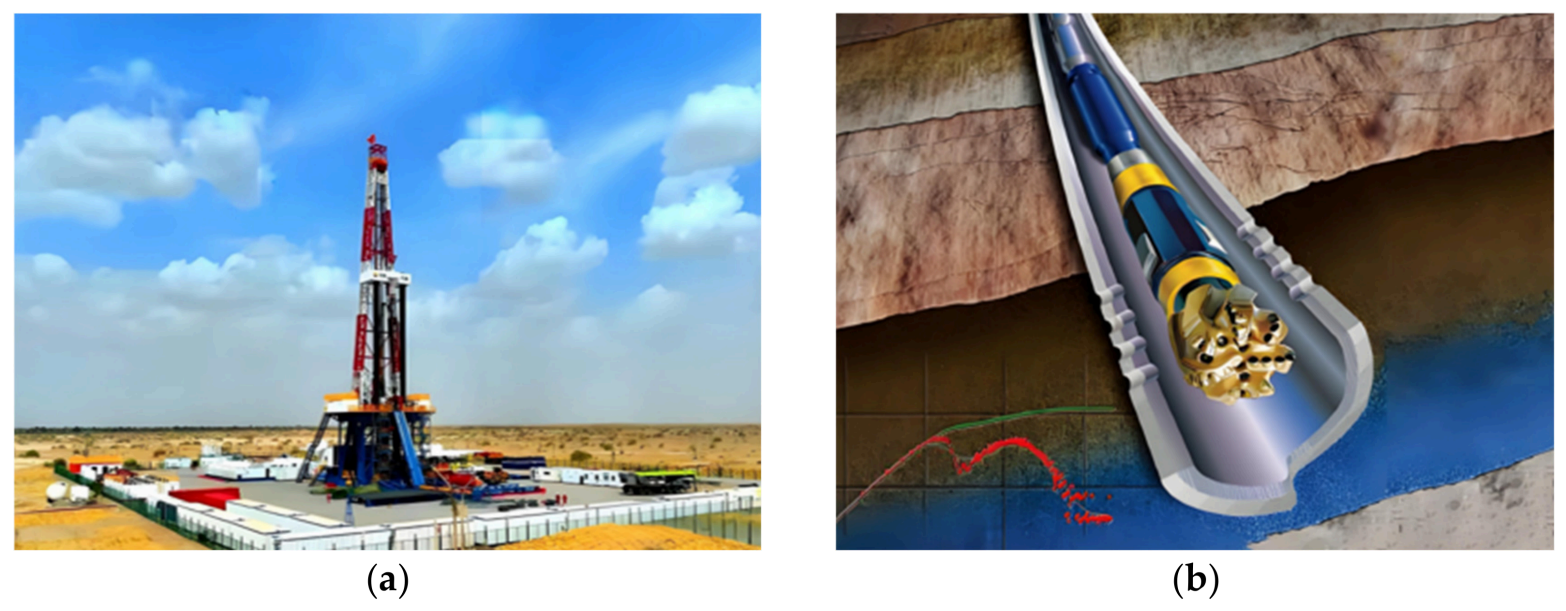
Deep-well drilling setup and downhole instrumentation: (**a**) 10,000 m deep drilling platform; (**b**) near-bit MWD sub.

Deep and ultra-deep well drilling is a fundamental technological tool for the exploration and development of deep hydrocarbon resources. As exploration and development move into deeper formations, drilling depths are constantly pushed to new records. Downhole temperature and pressure conditions, in turn, become increasingly severe (Figure 1a). Taking the Songliao Basin in China as an example, the geothermal gradient is approximately 4 °C/100 m, and the bottom-hole temperature of a 5000 m deep well can reach approximately 200 °C, while some ultra-deep wells may exceed 250 °C [1]. Under such high-temperature conditions, the drill bit parameter recorder is deployed into deep wells together with the drill bit (Figure 1b). The strain-gauge bridge, as the core sensing element, is significantly affected by temperature variations, resulting in output voltage drift with temperature, namely temperature drift. This drift causes the voltage signals of weight on bit (WOB) and torque received at the surface to deviate from the actual voltage values corresponding to the applied loads. Thermal expansion and elastic stretching can lengthen the drill string by more than 10 m during deep drilling. Temperature effects on downhole measurement accuracy should not be underestimated [2]. In recent years, physics-informed real-time correction methods have shown promise for ultra-deep well pressure and temperature sensing [3]. This progress further highlights the necessity of integrating physical priors into compensation models. In rotary punch drilling, impact loads couple the WOB and torque responses, which can be taken as a physical reference when downhole measurements are interpreted [4].

Traditional temperature-drift compensation methods fall into two main categories: hardware compensation and software compensation. Hardware compensation works by adding parts that react to thermal changes at the device, or building circuits with symmetrical paths to counteract the shift in data that thermal changes cause. This gives a good real-time response, but the precision of the method relies on how closely the electrical traits of the parts match. It is also difficult to cover the nonlinear drift under wide temperature conditions [5]. Software compensation uses the relationship between temperature and sensor output to build a model for correction during post-processing. It is cheap, and it is easy to adapt to different needs. Older methods for software compensation often relied on polynomial fitting, and lookup tables were also common. Downhole electromagnetic noise raises the background level, so those methods are effective only for temperature drift that is linear or weakly nonlinear. In deep wells, across a broad range of temperatures, the temperature drift in strain-gauge bridge output is hard to characterize. The output is strongly nonlinear.

In recent years, neural networks have been widely applied in the field of sensor drift compensation due to their powerful nonlinear mapping capabilities. Wei et al. [6] devised a temperature-drift compensation model for MEMS accelerometers. The model optimizes a backpropagation (BP) neural network with tent chaotic mapping and the sparrow search algorithm (SSA). This approach reduced the mean absolute percentage error from 2% to 0.2%. Separately, Wang et al. [7] combined gray wolf optimization (GWO) with a kernel extreme learning machine for pressure sensor temperature compensation. Other researchers applied particle-swarm-optimization-based extreme learning machines to compensate resonant MEMS electric-field sensors, reducing the maximum relative drift by approximately 78.8% compared with conventional methods [8]. For high-temperature measurement-while-drilling (MWD) directional inclinometers, Yang et al. [1] designed a dynamic temperature compensation strategy using wavelet-based filtering and multiscale analysis. At 150 °C, the azimuth accuracy reached ±2° or better. Separately, a temperature compensation approach for MEMS gyroscopes was developed that combines an improved complete ensemble empirical mode decomposition with an optimized extreme learning machine [9], and BP neural-network-based temperature drift compensation for electrostatically suspended accelerometers has also achieved promising results [10]. The extreme learning machine (ELM) has attracted increasing attention in sensor compensation due to its high training efficiency. However, conventional ELM is sensitive to hyperparameter selection. Recently, physics-informed extreme learning machine (PIELM) and its improved variants have been proposed, including multi-region tagged PIELM for efficient thermal analysis [11] and an ELM–AdaBoost ensemble strategy for temperature error compensation of fiber-optic gyroscopes [12]. The application of multilayer nonlinear local receptive field ELM in geological logging gas analysis further demonstrates the applicability of ELM variants in geological and drilling engineering fields [13]. However, these methods are essentially data-driven models, and their predictions depend entirely on the quality and coverage of the training data. In deep-well drilling, downhole measurements generally lack corresponding ground-truth load labels. Laboratory calibration experiments, however, cannot readily reproduce the high-temperature and high-pressure conditions found downhole. The combination of limited samples and insufficient operating-condition coverage poses substantial challenges to the generalization capability of purely data-driven models in downhole applications. To address this issue, previous studies have incorporated physical constraints into ELM, using a multi-region labeling strategy to efficiently solve multi-medium heat conduction problems [14].

The development of physics-informed neural networks (PINNs) creates a different method for research. It functions outside the constraints that exist in techniques based only on collected observations [15]. The network integrates the residuals of physical equations into the loss function, which ensures that the results of the network align with the observed data and the requirements of physics. By using this method, the models follow the laws of physics when the available information contains gaps or noise. In recent times, researchers have improved PINNs and related models in thermal applications, such as temperature field reconstruction and inverse heat conduction problems [16,17]. However, PINNs usually use architectures where every neuron connects to every other neuron in the next layer. The training process requires the system to adjust internal values repeatedly through backpropagation, which causes a large requirement for processing power and time. ELM is a feedforward neural network that contains one layer between the input and output. Its input weights and biases are chosen by a random process and remain constant. Only the output weights require a mathematical solution. The training speed is much greater than the speed of standard neural networks [18]. Embedding physical information into the ELM framework—namely PIELM—not only retains the efficient training advantage of ELM, but also incorporates physical constraints into the solution process of output weights by augmenting the linear system [19,20]. The PIELM architecture gives accurate results in several areas of solid mechanics, and those areas include how solid objects deform, how materials break, and how structures move when forces change over time [21,22,23]. In particular, Mishra et al. [23] developed a PIELM framework that uses null-space projection. The method was successfully applied to static bending of plates, showing that the algorithm meets the requirements for mathematical continuity. Separately, Dwivedi and Srinivasan [24] set out the first methods for using PIELM to find solutions for partial differential equations. These properties make PIELM particularly useful for engineering tasks where only a few data points exist and the physical laws are well known. It is a new tool that corrects the thermal movement of drill bits in deep wells. The model is fast when it calculates, and it follows physical principles. This method, which uses physics, is also relevant to subjects outside mechanical engineering. In one case, a model was used to assess how rubber mixes in a closed system [25] and analyze how hydraulic systems manage force [26]. To improve management, scientists used physical data fusion to model soil and water in agriculture [27,28]. Those studies show that machine learning is more effective when it includes physical constraints.

The efficiency of PIELM is a result of the values assigned to its hyperparameters—those variables are the count of hidden neurons, the constant for regularization, and the coefficients for physical constraints. The adjustment of parameters by hand within a space of six dimensions is slow. It also does not guarantee that the results represent the most effective possible configuration. Swarm intelligence optimization algorithms do not need information about gradients and possess the ability to search large solution spaces. The presence of these characteristics is the reason researchers frequently use them to set the hyperparameters of neural networks automatically [29]. EEFO is a swarm intelligence algorithm that researchers developed recently. By simulating how electric eels forage through interaction, resting, hunting, and migration, the algorithm maintains a proportion between wide searching and focused refinement [30]. However, the standard version of EEFO remains difficult to use for non-convex optimization in many dimensions. The variety within the population is lower as the process repeats and the accuracy of local searches is less during the final steps [31]. Several improvement strategies have been proposed to address these limitations, such as incorporating quasi-opposition-based learning and trigonometric mutation operators [32,33]. In addition, improved EEFO algorithms have been applied to electric vehicle design optimization [34] and distributed power network reconstruction [35], demonstrating their potential in engineering optimization problems. Furthermore, the open-source unified PIELM framework developed by Struniawski et al. [36] provides a convenient basis for integrating PIELM with optimization algorithms.

In summary, a compensation method for deep-well drill bit temperature drift is developed using an improved EEFO algorithm combined with PIELM. The contributions of this study lie in the compensation framework, the quantified role of the hyperparameter optimization, and the validation methodology for the no-ground-truth downhole scenario:

1. A PIELM model incorporating four weak physical constraints, including trend consistency, regression deviation, proportional relationship, and physical process continuity, is developed. The trend and regression priors associated with drill bit temperature drift are embedded into the output-weight solving process in the form of an augmented linear system.
2. An improved EEFO (IEEFO) is constructed as the search engine for PIELM hyperparameter optimization, and an equal-budget ablation protocol is established to determine the deployed configuration: dynamic-boundary quasi-opposition-based learning is the most effective single strategy, and the t-distribution mutation is excluded because it is significantly worse than standard EEFO; the final configuration attains the lowest mean fitness among the five configurations on the target problem and significantly outperforms the initial three-component version on the benchmarks. Multiple independent runs on the target problem show that the accuracy gain originates from the hyperparameter optimization itself rather than from the choice of optimizer.
3. A multi-source dataset covering 25 °C to 120 °C is assembled from calibration experiments and thermo-mechanical coupled simulations; for the downhole assessment, where no load ground truth exists and the references can only be derived from the voltage signals, a consistency-verification framework is established that combines four affine mapping protocols, a domain-coverage analysis, and physical-constraint checks; the framework reveals that the apparent drift-elimination rate originates from the mapping protocol rather than from a reduction in measurement error, so quantitative accuracy claims are restricted to the calibration domain.

The rest of this paper is organized as follows. Section 2 describes the model framework, the fundamentals of PIELM, the four physical constraints, and the improved EEFO. Section 3 describes how the dataset was assembled, the hyperparameter settings, and the accuracy reached during training. Section 4 processes the downhole data and validates the model along two dimensions: the error-temperature correlation and the satisfaction of the physical constraints. Section 5 summarizes the findings and outlines future work.

## 2. Model Framework and Theoretical Methodology

### 2.1. Temperature Drift Compensation Model Framework

**Figure 2:**
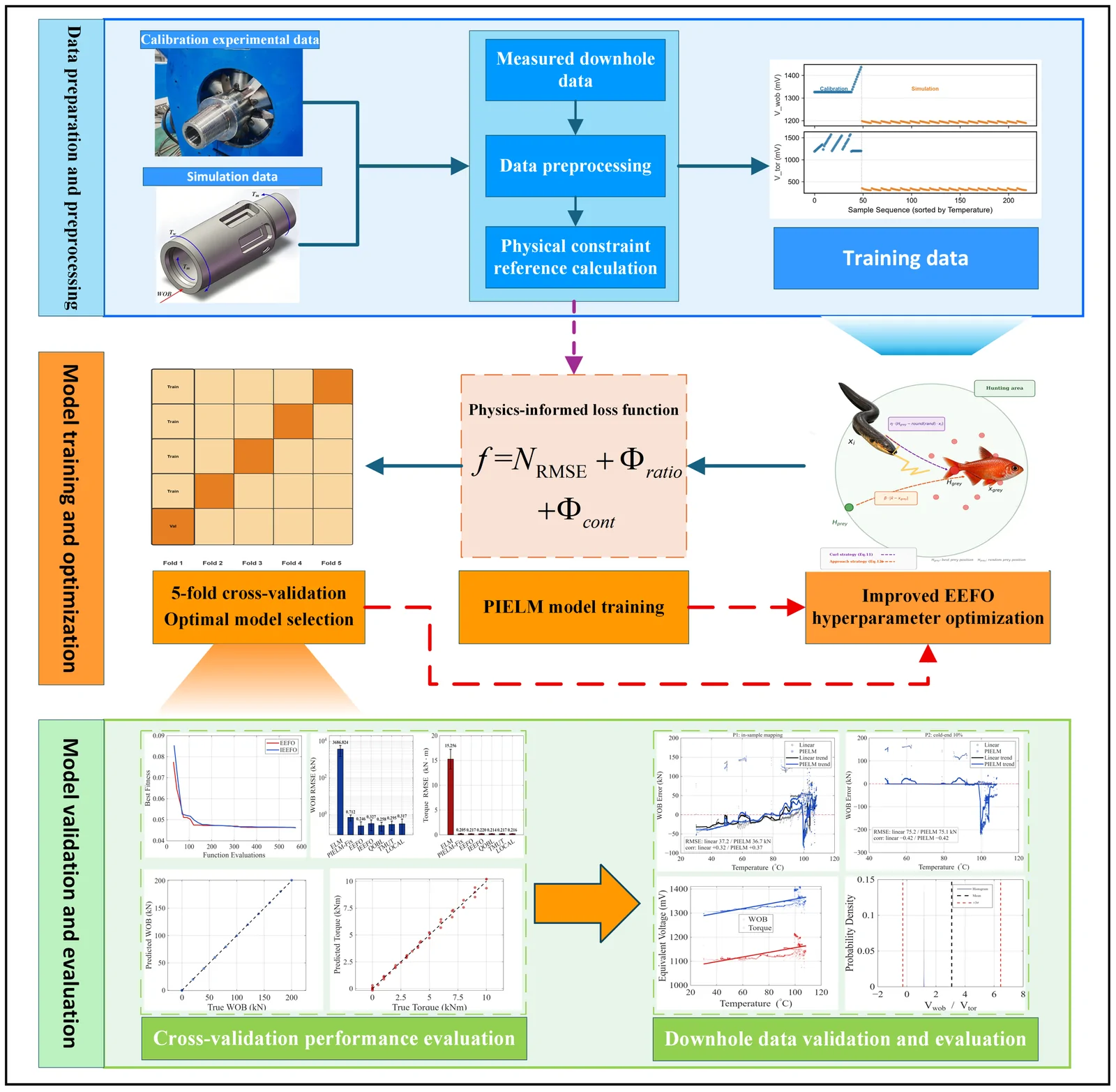
Framework and implementation process of the temperature drift compensation method.

The overall framework of the proposed temperature-drift compensation method is shown in Figure 2. The method aims at downhole drill-bit temperature-drift compensation: calibration and simulation data are used to establish the temperature–voltage–force mapping, and physical priors are extracted from the measured downhole voltage statistics as constraints, so that the model outputs physically consistent and temperature-decoupled compensated WOB and torque under downhole conditions. The overall implementation proceeds as follows:

1. First, the calibrated experimental data are mixed with the simulation data and normalized to form the training set. Three quantities from the downhole voltage data are extracted for the physical constraint benchmarks, including the temperature-correlation signs, voltage ratio statistics, and rate-of-change thresholds.
2. Next, with PIELM adopted as the core model, physical constraints are embedded into the training objective through the augmented output-weight solving process and penalty terms. The improved EEFO algorithm is utilized to automatically search for the optimal hyperparameter configuration through a five-fold cross-validation strategy.
3. Finally, the model accuracy and stability are evaluated in the training domain by using cross-validation metrics. The trained model is then applied to the measured downhole data, and the consistency of the compensated output is assessed through multi-mapping-protocol comparisons and physical-constraint checks.

### 2.2. Physics-Informed Extreme Learning Machine

ELM is a training algorithm for hidden-layer feedforward neural networks. Its core feature is that the weights and biases between the input layer and the hidden layer are randomly generated and then remain constant [18]. Only the output-layer weights need to be analytically solved through a linear system, eliminating the requirement for iterative backpropagation and therefore achieving extremely high training efficiency.

Assuming that there are *N* training samples ${\left(x_{i},y_{i}\right)}_{\left(i=1\right)}^{N}$, where *x_i_* ∈ *R^n^* represents the input vector and *y_i_* ∈ *R^m^* represents the corresponding output vector, an ELM network with *L* hidden neurons can be expressed as:

(1) $$y_{i}=\sum_{j=1}^{L}\beta_{j}g\left(\omega_{j}\cdot x_{i}+b_{j}\right),i=1,\ldots ,N$$

The activation function is written as *g*(*x*). The quantities *ω_j_*, *b_j_*, and *β_j_* are, respectively, the input weight, bias, and output weight associated with the j-th hidden neuron. These equations can be arranged as the following matrix form:

**Hβ** = **Y** (2)

Here, **H** denotes the hidden-layer output matrix, **β** the output weight matrix, and **Y** the target matrix. The output weights are analytically solved via the Moore–Penrose pseudoinverse as:

(3) $$\beta =H^{†}Y={\left(H^{T}H\right)}^{-1}H^{T}Y$$

The generalized inverse of the hidden-layer output matrix is denoted by **H**^†^.

Although ELM provides extremely fast training, its purely data-driven nature prevents it from utilizing known physical laws within the system. To overcome this limitation, PIELM introduces physical constraints into the output-weight solving process in the form of additional equations [19]. The fundamental idea of PIELM is to extend the conventional least-squares framework of ELM by introducing additional rows corresponding to physical constraints into the linear system, enabling the network output to simultaneously satisfy both data fitting requirements and physical laws.

For a set of linear physical constraints, residual points are selected within the computational domain, and corresponding constraint operators are applied to the network output. The physical constraints can be formulated as an augmented linear system:

(4) $$\left[\begin{array}{c} H\\ \sqrt{\lambda }L\left(H\right) \end{array}\right]\beta =\left[\begin{array}{c} Y\\ \sqrt{\lambda }ƒ \end{array}\right]$$

where *L* represents the linear constraint operator, *f* denotes the corresponding right-hand-side term, and *λ* represents the weighting coefficient of the physical constraint. By introducing the augmented matrix **H***_aug_* and augmented target matrix **Y***_aug_*, the output weights can be further obtained as:

(5) $$\beta ={\left(H_{\mathrm{aug}}^{T}H_{\mathrm{aug}}\right)}^{-1}H_{\mathrm{aug}}^{T}Y_{\mathrm{aug}}$$

### 2.3. Construction of Four Weak Physical Constraints

To improve the temperature drift compensation performance of deep-well drill bit measurements, the relationships among temperature, WOB voltage, and torque voltage are represented by four weak physical constraints and incorporated into the physics-informed extreme learning machine. Because the PIELM output layer produces loads whereas the constraints are formulated in the voltage domain, all constraint operators act on the network output through the affine voltage-load calibration map *V* = (*F* − *d*_0_)/*k*_0_, where F denotes the load output of the PIELM (WOB or torque), V the equivalent voltage recovered from the load, and *k*_0_ and *d*_0_ the slope and intercept of the voltage-load calibration line *F* = *k*_0_*V* + *d*_0_ fitted on the training fold. Among the constraints, the trend consistency and regression deviation constraints are linear in the output weights and are embedded into the output-weight solution through an augmented linear system; in this paper, this class is termed embedded constraints, a name chosen for how the constraints are imposed rather than for the exact satisfaction associated with hard constraints in the literature. The proportional relationship and continuity constraints are one-sided and nonlinear and are imposed as penalty terms in the fitness function; this class is termed soft constraints. This arrangement allows the model outputs to remain consistent with the observed physical characteristics of the drilling system.

#### 2.3.1. Trend Consistency Constraint

During drilling operations, temperature variations generally drive the WOB and torque voltage signals in a definite direction. To preserve the physical trend observed in historical measurements, the predicted voltage variations are required to follow the same correlation direction as the historical data; predictions exhibiting an opposite trend are penalized. The constraint is implemented as a linear pair-difference operator so that it can be embedded into the augmented linear system.

Let h(T) denote the hidden-layer output row of the network at temperature T, so that a predicted value is *y*(*T*) = *h*(*T*)·*β*. From the N training samples, up to min(30, N) index pairs (i, j) are drawn at random with |*T_i_* − *T_j_*| > 0.2Δ*T*, so that only pairs spanning a substantial temperature gap contribute. Each valid pair requires the prediction difference to carry the historical correlation direction s (*s* = ±1, taken from the sign of the temperature-signal correlation of the uncompensated measurements) with a magnitude proportional to the normalized temperature gap:

(6) $$s\cdot \left(\;y\left(T_{i}\right)-\;y\left(T_{j}\right)\;\right)\;=\;\frac{\left|T_{i}-\;T_{j}\right|}{\Delta T}$$

Because *y*(*T*) = *h*(*T*)·*β* is linear in the output weights, the above is a system of linear equations in β. Let P be the *N_v_* × *L* matrix whose p-th row is *h*(*T_i_*) − *h*(*T_j_*) for the p-th valid pair, and let t be the vector with entries $\frac{\left|T_{i}-T_{j}\right|}{\Delta T}$; the trend block appended to the linear system is then

(7) $$\left[\;\begin{array}{c} \;H_{\mathrm{aug}}\\ \sqrt{\frac{\lambda_{\mathrm{trend}}}{N_{v}}}\cdot Q \end{array}\;\right]\cdot \beta \approx \left[\begin{array}{c} Y_{\mathrm{aug}}\\ \sqrt{\frac{\lambda_{\mathrm{trend}}}{N_{v}}}\cdot s\cdot t \end{array}\;\right]$$

where *λ_trend_* weights this constraint. A correlation-based operator would be nonlinear in β and could not enter the augmented linear system; the pair-difference operator above preserves the intended directional information while remaining linear.

#### 2.3.2. Regression Deviation Constraint

The voltage responses of WOB and torque generally vary with temperature, and their historical data can be used to fit a regression model such as a quadratic polynomial. Based on this observation, a regression deviation constraint is introduced to prevent the predicted voltages from deviating excessively from the historical temperature–voltage relationship. This constraint reflects the physical assumption that temperature-induced voltage drift generally varies smoothly with temperature rather than changing abruptly [24].

Based on the historical calibration data, quadratic regression models are established for the WOB and torque voltage channels as functions of temperature:

*P_base_*(*T*) = *a*_2_*T*^2^ + *a*_1_*T* + *a*_0_,*M_base_*(*T*) = *b*_2_*T*^2^ + *b*_1_*T* + *b*_0_ (8)

For the current predicted voltage and the corresponding temperature, calculate the normalized residuals:

(9) $$z_{P,i}=\frac{P_{i}-P_{\mathrm{base}}\left(T_{i}\right)}{\sigma_{P}},z_{M,i}=\frac{M_{i}-M_{\mathrm{base}}\left(T_{i}\right)}{\sigma_{M}}$$

Two standard deviations are defined from the historical voltage data. One is *σ_P_* for the WOB channel, and the other is *σ_M_* for the torque channel.

Rather than being treated as a separate penalty, the regression deviation is imposed through additional rows of the augmented linear system:

(10) $$\left[\;\begin{array}{c} H_{\mathrm{aug}}\\ \sqrt{\lambda_{\mathrm{reg}}\cdot c_{r}}\cdot H \end{array}\;\right]\cdot \beta \;\approx \;\left[\;\begin{array}{c} Y_{\mathrm{aug}}\\ \sqrt{\lambda_{\mathrm{reg}}\cdot c_{r}}\cdot \left(w\circ {\tilde{y}}_{\mathrm{reg}}\right) \end{array}\;\right]$$

where *λ_reg_* weights this block, and *c_r_* equals 1.0 for the WOB channel and 0.3 for the torque channel, reflecting the weaker temperature dependence of the torque channel; *w* is the square root of the sample weight vector, and ${\tilde{y}}_{\mathrm{reg}}$ denotes the quadratic regression defined above. This term keeps the model output aligned with the smooth drift curve derived from historical data and suppresses abnormal fluctuations.

#### 2.3.3. Proportional Relationship Constraint

Torque and WOB are coupled during normal drilling operations. Therefore, the voltage ratio should be maintained in a reasonable range. If predictions deviate significantly from the historical normal range, penalties should be imposed.

The voltage ratio of each sample is calculated as:

(11) $$r_{i}=\frac{P_{i}}{M_{i}+\varepsilon }$$

where ε is a small positive constant only to avoid numerical overflow when the denominator approaches zero, without affecting the calculation accuracy. The mean value *μ_r_* and standard deviation *σ_r_* of the ratio *r* are calculated from historical data after removing obvious outliers. The normalized deviation is introduced as:

(12) $$d_{i}=\frac{\left|r_{i}-\mu_{r}\right|}{\sigma_{r}}$$

Penalties apply only to deviations exceeding 3σ, with a squared term introduced to penalize larger departures more severely:

(13) $${\mathrm{viol}}_{\mathrm{ratio},i}=\max\left(0,d_{i}-3\right),R_{\mathrm{ratio}}=\frac{1}{N}\sum_{i=1}^{N}{\mathrm{viol}}_{\mathrm{ratio},i}^{2}$$

The proportional relationship penalty is:

Φ*_ratio_* = *λ_ratio_*·log(1 + *R_ratio_*) (14)

This design permits the ratio to fluctuate freely within ±3σ and penalizes outliers progressively—a choice consistent with engineering tolerance practice. The penalty enters the fitness function through the logarithmic transform *λ_ratio_*·log(1 + *R_ratio_*), which keeps its scale comparable to the root-mean-square error (RMSE) term.

#### 2.3.4. Physical Process Continuity Constraint

During the drilling process, temperature, WOB, and torque are generally continuous. Abrupt changes do not occur in these signals. If a sharp voltage fluctuation is predicted, penalties should be imposed.

Assuming a constant sampling interval Δ*t*, the voltage variation rates are calculated as:

(15) $${\dot{P}}_{i}=\frac{\left|P_{i+1}-P_{i}\right|}{\Delta t},{\dot{M}}_{i}=\frac{\left|M_{i+1}-M_{i}\right|}{\Delta t},i=1,\ldots ,N-1$$

From the historical data, the 95th percentiles of the change rates of the WOB voltage and the torque voltage are calculated and used as the thresholds *τ_P_* and *τ_M_*. For the values exceeding the threshold, the relative excess is defined as:

(16) $$u_{P,i}=\max\left(\frac{{\dot{P}}_{i}-\tau_{P}}{\tau_{P}}\right),u_{M,i}=\max\left(\frac{{\dot{M}}_{i}-\tau_{M}}{\tau_{M}}\right)$$

The continuity violation is quantified using the mean squared excess,

(17) $$C_{\mathrm{wob}}=\frac{1}{N-1}\sum_{i=1}^{N-1}u_{p,i}^{2},C_{\mathrm{tor}}=\frac{1}{N-1}\sum_{i=1}^{N-1}u_{M,i}^{2}$$

The physical process continuity penalty is expressed as

(18) $$\Phi_{\mathrm{cont}}=\lambda_{\mathrm{cont}}\cdot \log\left(1+\frac{C_{\mathrm{wob}}+C_{\mathrm{tor}}}{2}\right)$$

Here, *λ_cont_* denotes the weighting coefficient of the continuity constraint. This penalty term encourages the model to output a smooth voltage sequence and suppresses non-physical step changes [23].

The proportional-relationship and continuity penalties enter the fitness function together with the prediction error, while the trend consistency and regression deviation constraints act inside the output-weight solve. The fitness function is defined as the weighted sum of the prediction error and the two physical penalty terms,

*f* = *N_RMSE_* + Φ*_ratio_* + Φ*_cont_* (19)

where the data-fitting term is dimensionless: $N_{\mathrm{RMSE}}=0.5\left(\frac{{\mathrm{RMSE}}_{\mathrm{wob}}}{\sigma_{\mathrm{wob}}}+\frac{{\mathrm{RMSE}}_{\mathrm{tor}}}{\sigma_{\mathrm{tor}}}\right)$, with σ being the standard deviation of each target channel on the training fold.

### 2.4. Improved Electric Eel Foraging Optimization Algorithm

#### 2.4.1. Electric Eel Foraging Optimization Algorithm

The electric eel foraging optimization (EEFO) algorithm is a recently developed swarm intelligence method. It is inspired by the collective foraging behavior of electric eels in nature [29]. Electric eels can generate weak electric fields for environmental sensing and communication among individuals, while strong electric discharges are used to immobilize prey. During collective hunting, electric eels exhibit four behavioral patterns, including interaction, resting, hunting, and migration. EEFO uses mathematical modeling to simulate the above behavior and has constructed an adaptive switching mechanism between global exploration and local exploitation.

1. Interaction Behavior

Electric eels perform large-scale exploration through information exchange among individuals. Each electric eel randomly selects another individual within the population or a random position in the search space for position updating, while introducing a random perturbation vector **C** representing environmental disturbance. The updating rule is given by:

(20) $$x_{i,\mathrm{new}}=\left\{\begin{array}{c} x_{j}+C\cdot \left(\bar{x}-x_{i}\right),f\left(x_{j}\right)<f\left(x_{i}\right),p_{1}>0.5\\ x_{j}+C\cdot \left(x_{r}-x_{i}\right),f\left(x_{j}\right)<f\left(x_{i}\right),p_{1}\leq 0.5\\ x_{i}+C\cdot \left(\bar{x}-x_{j}\right),f\left(x_{j}\right)\geq f\left(x_{i}\right),p_{1}>0.5\\ x_{i}+C\cdot \left(x_{r}-x_{j}\right),f\left(x_{j}\right)\geq f\left(x_{i}\right),p_{1}\leq 0.5 \end{array}\right.$$

The symbol *x_j_* denotes a randomly selected eel from the population. The term $\bar{x}$ refers to the centroid position of the entire group, and *x_r_* denotes a position sampled randomly from the search space. *p*_1_ follows a uniform distribution over (0,1).

2. Resting Behavior

In resting behavior, a resting region is determined before the position is updated. The position of this region is based on projecting the current position onto the main diagonal of the normalized search space. The resting position is calculated as:

(21) $$\begin{array}{c} R_{i}\left(t+1\right)=Z\left(t\right)+\alpha \cdot \left|Z\left(t\right)-x_{\mathrm{prey}}\left(t\right)\right|\\ v_{i}\left(t+1\right)=R_{i}\left(t+1\right)+n_{2}\cdot \left(R_{i}\left(t+1\right)-\mathrm{round}\left(\mathrm{rand}\right)\cdot x_{i}\left(t\right)\right) \end{array}$$

where *Z*(*t*) is the center of the resting region, *α* denotes a scale factor that decreases with iteration, and *n*_2_ represents a standard normal distribution.

3. Hunting Behavior

During hunting, the eels approach the prey by creating an “electric field circle” and gradually narrowing the search region before the final attack. The mathematical model of this behavior is expressed as:

(22) $$\begin{array}{c} H_{\mathrm{prey}}\left(t+1\right)=x_{\mathrm{prey}}\left(t\right)+\beta \cdot \left|\bar{x}\left(t\right)-x_{\mathrm{prey}}\left(t\right)\right|\\ v_{i}\left(t+1\right)=H_{\mathrm{prey}}\left(t+1\right)+\eta \cdot \left(H_{\mathrm{prey}}\left(t+1\right)-\mathrm{round}\left(\mathrm{rand}\right)\cdot x_{i}\left(t\right)\right) \end{array}$$

where *β* represents the scale factor of the hunting region and *η* denotes the curling factor.

4. Migration Behavior

When electric eels sense their prey, they will move from their resting area to the hunting area. A Lévy-flight term is included in the position update to provide additional long-range search capability and reduce the possibility of remaining around a local optimum:

*v*_i_(*t* + 1) = −*r*_5_·*R_i_*(*t* + 1) + *r*_6_·*H_r_*(*t* + 1) − *L*·(*H_r_*(*t* + 1) − *x_i_*(*t*)) (23)

where *H_r_* denotes a position sampled within the hunting region. The quantities *r*_5_ and *r*_6_ are uniformly distributed random numbers in (0,1). The term *L* denotes the step length generated by Lévy flight.

5. Behavioral Switching Mechanism

The selection of exploration or exploitation behavior in EEFO depends on the energy factor *E*(*t*). Its value is calculated by:

(24) $$E\left(t\right)=4\cdot \sin\left(1-\frac{t}{T_{\max}}\right)\cdot \ln\left(\frac{1}{r_{7}}\right)$$

Here, *t* denotes the current iteration, *T*_max_ is the maximum number of iterations, and *r*_7_ is a random number in the range (0,1). When *E*(*t*) > 1, the algorithm performs global exploration through interaction behavior. When *E*(*t*) ≤ 1, it switches to local exploitation behaviors, including migration, resting, and hunting. The energy factor exhibits an overall decreasing oscillation trend as iterations increase, enabling a natural transition from exploration to exploitation.

#### 2.4.2. Improvement Strategies

To address the limitations of the standard EEFO for high-dimensional non-convex problems, i.e., the gradual loss of population diversity during iteration and insufficient search accuracy in the final stage [32], this study improves the original EEFO in two aspects, population diversity preservation and final optimization accuracy enhancement.

1. Dynamic-Boundary Quasi-Opposition-Based Learning

The conventional quasi-opposition-based learning (QOBL) strategy employs fixed global search boundaries. However, after population convergence in the later optimization stage, the opposite solutions generated by the fixed boundaries are usually located far away from the current population distribution. Therefore, these solutions are unlikely to be selected through greedy competition, reducing the effectiveness of the opposition-based learning strategy. To address this problem, a dynamic-boundary strategy is proposed, where a gradually shrinking hybrid boundary is introduced instead of fixed global boundaries:

(25) $$X_{i}^{\left(\mathrm{qobl}\right)}=\left[\beta_{t}\cdot \mathrm{lb}+\left(1-\beta_{t}\right)L_{\mathrm{dyn}}\right]+\left[\beta_{t}\cdot \mathrm{ub}+\left(1-\beta_{t}\right)U_{\mathrm{dyn}}\right]-\gamma X_{i}$$

Two boundary values are involved: lb marks the lower limit and ub marks the upper limit of the search space. $\beta_{t}=0.6\left(\frac{1-t}{T_{\max}}\right)$ controls the boundary contraction rate, while *L_dyn_* and *U_dyn_* represent the actual distribution range of the current population in each dimension. *γ* ~ U (0.1, 1.0) is a random perturbation factor. This strategy is only applied to the bottom 25% of individuals ranked by fitness value. Consequently, the additional evaluation number is reduced from PopSize times per iteration to [PopSize/4] times, improving computational efficiency.

2. Local Fine Search Strategy

After the main iterative optimization process, a step-adaptive fine-search strategy is applied to the global optimal solution to improve the final optimization accuracy:

*X*_cand_ = *X*_prey_ + *δ*(ub − lb) · *N*(0,*I*) (26)

(27) $$\delta_{k+1}=\min\left(1.35_{k}^{I}\cdot 0.6_{k}^{1-I}\cdot \delta_{k},0.05\right)$$

The initial search step size *δ*_0_ is set to 1% of the search-space range; after each accepted candidate, the step size is expanded by a factor of 1.35 (capped at 5% of the range), whereas after a rejected candidate it is contracted by a factor of 0.6. A total of 20 refinement steps are performed, and each step requires only one fitness evaluation. Here, *I_k_* is the acceptance indicator: *I_k_* = 1 when a candidate improves the current best, and *I_k_* = 0 otherwise.

#### 2.4.3. Equal-Budget Evaluation and Budget Alignment

The dynamic-boundary QOBL adds *k* = max(3,[PopSize/4]) candidate evaluations per iteration, and the local fine search adds 20 final evaluations, so IEEFO consumes 575 function evaluations over 30 iterations, whereas standard EEFO consumes only 465 over the same iteration count—a 24% advantage that would make any comparison at equal iteration counts unfair. All comparisons in this work therefore use an equal function-evaluation (FE) budget: the iteration count of each algorithm is derived from its per-iteration evaluation cost, and the allowance of the final fine search is set in proportion to the budget (20 evaluations at FE = 575). The actual evaluation counts are recorded and reported in all comparisons.

## 3. Dataset Construction and Model Training

### 3.1. Temperature Calibration Experiment

**Figure 3:**
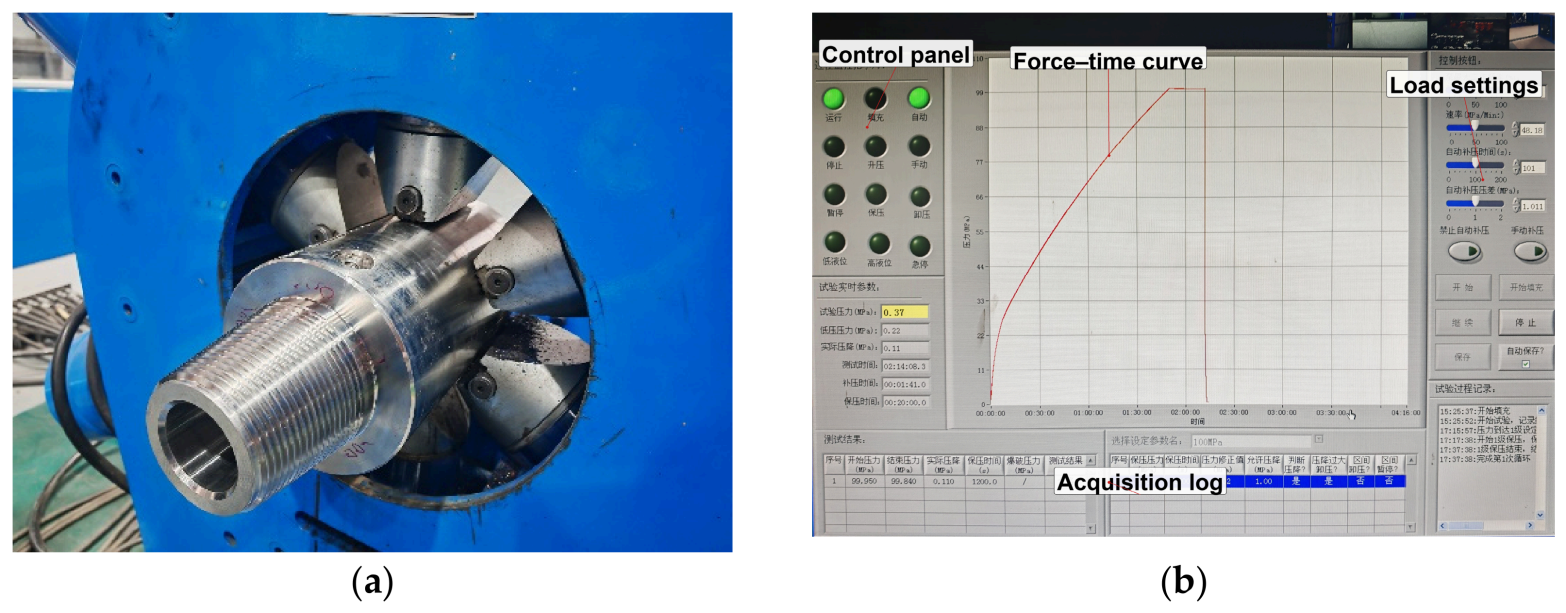
Calibration experimental setup and data acquisition system: (**a**) hydraulic loading system; (**b**) data acquisition system.

The measured downhole data contain only three channels of time-series signals, namely temperature *T*, WOB voltage *V*_wob_, and torque voltage *V*_tor_, without corresponding load labels for WOB and torque. Therefore, these data cannot be directly used for supervised training of the temperature drift compensation model. For this purpose, calibration experiments were performed under ambient conditions (25 ± 2 °C). The recorder was subjected to known mechanical loads while the bridge output voltages were synchronously acquired, and the resulting data served to construct a quantitative relationship between voltage and load. The experimental setup is illustrated in Figure 3.

The hydraulic loading system applied WOB and operating torque in two separate loading sequences (one factor at a time), with a loading accuracy better than ±0.5% full scale (FS). WOB was swept through 11 discrete levels from 0 to 200 kN in 20 kN increments, yielding one valid steady-state sample per nonzero level. The operating torque was swept through 10 levels within the range of 0 to 10 kN·m at a step size of 1 kN·m. For each nonzero level, the measurement was approximately repeated 3 times. In total, 36 sets of valid torque samples were obtained at zero WOB. The two factors were applied in separate sequences, so no simultaneous WOB–torque combination was measured in the calibration experiments. Strain gauges were pre-attached inside the sensor mounting grooves and configured into a Wheatstone full-bridge circuit. The voltage signals were sampled at a 1 kHz rate after conditioning, amplification, and filtering. Each load condition was maintained for 30 s, and the steady-state average value was calculated. Through this procedure, a total of 49 valid calibration samples were obtained, each consisting of an input vector and the corresponding output load vector. Based on these samples, the linear voltage-load regressions fitted on these samples attain *R*^2^ = 0.99984 for WOB (n = 10) and *R*^2^ = 0.99393 for torque (n = 36).

### 3.2. Thermo-Mechanical Coupled Simulation

**Figure 4:**
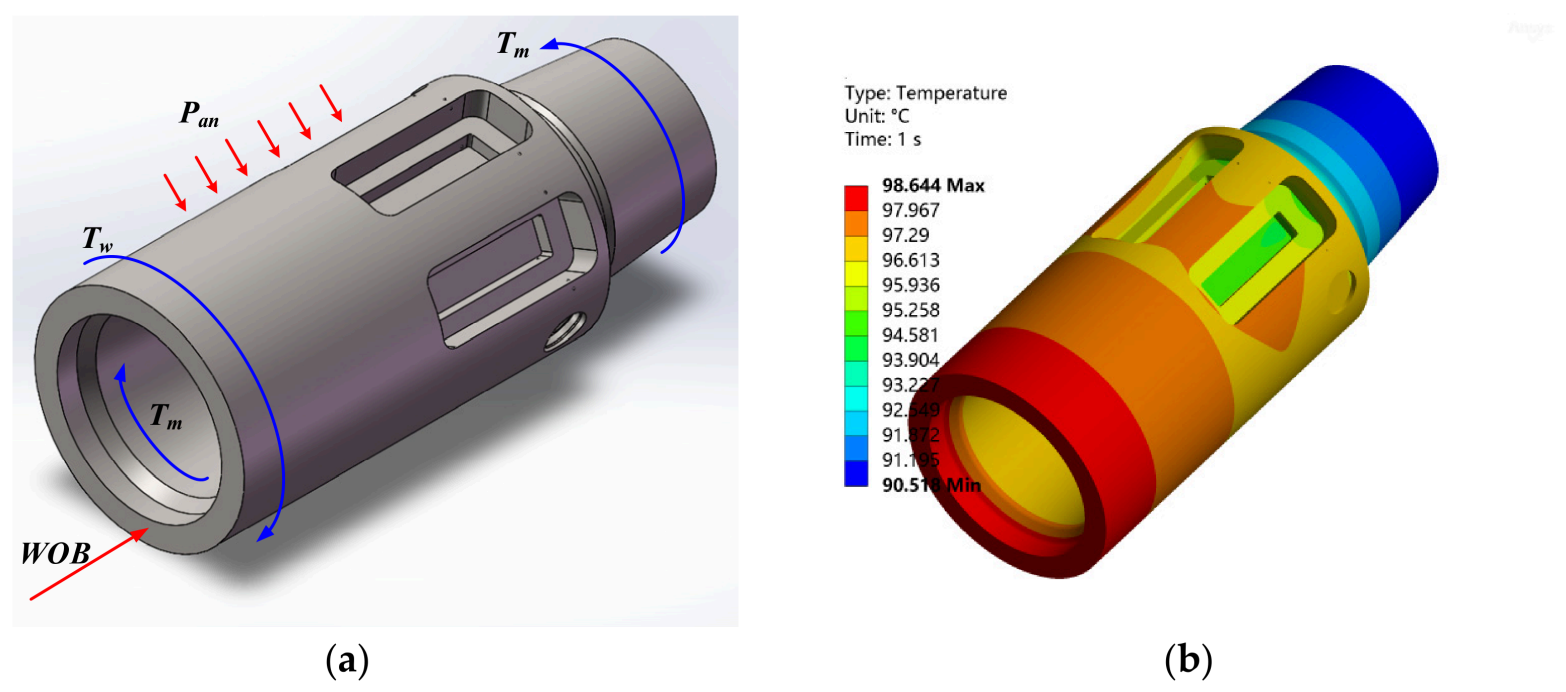
Simulation results of the drill bit parameter recorder: (**a**) force analysis of drill bit parameter recorder; (**b**) temperature distribution under different temperatures.

The ground calibration experiment was conducted at room temperature, so the samples contain no temperature-dependent information; additional high-temperature samples were generated by thermo-mechanical coupled finite element analysis of the drill bit parameter recorder, as illustrated in Figure 4. The recorder is made of 15–15 HS MAX nitrogen-strengthened austenitic stainless steel, with temperature-dependent thermal expansion and elastic modulus, and is solved in ANSYS 2022 R2 with a sequential coupling scheme: a steady-state thermal solution provides the temperature field, which is mapped as body loads into a static structural analysis, with convective heat transfer to the drilling fluid applied on the external surface. The mesh is locally refined around the strain-gauge grooves with a global element size of 3 mm and 1 mm at the groove surface, and a five-level grid-independence study selects a 523,918-element model; full modeling details are given in the companion study [37]. Seventeen ambient temperatures between 50 °C and 120 °C were considered. The mechanical loading followed ten representative drilling states in which WOB and torque co-vary, with 0–200 kN corresponding one-to-one to 0–10 kN·m, so the loading space has one degree of freedom rather than an orthogonal two-factor design; the magnitude, position, and direction of each load follow the actual assembly configuration and stress state of the recorder during drilling.

Virtual probes were placed at the strain-gauge installation locations. The normal elastic strain along X, which is sensitive to WOB, and the shear strain in XY, which is sensitive to torque, were extracted; the 17 temperatures and 10 load states give 170 cases, which were converted into equivalent voltages using the strain-voltage conversion coefficients established in the calibration experiments. A two-way variance decomposition of the resulting voltage columns shows that the load state explains 99.97% of the WOB-voltage variance and 98.22% of the torque-voltage variance, whereas the temperature main effect accounts for only 0.00% and 0.41%; the simulated voltages thus carry little temperature-drift information, and the simulation set serves primarily to extend the operating-condition coverage and to regularize the model, its influence being quantified by the weight sensitivity analysis in Section 3.3. In addition, the voltage scale obtained from the strain-voltage conversion differs from the calibration scale. For the WOB channel, the fitted slopes are −0.0419 and +0.5464 mV/kN, so the simulation samples are used as auxiliary data with reduced weighting, and quantitative claims are anchored to the calibration scale, which defines the absolute voltage reference. The simulation data inform the shape of the temperature dependence, whereas quantitative transfer beyond the calibration domain relies on the measured calibration relation. The simulation contribution is therefore best regarded as a physics-based regularizer; validated high-temperature evidence would require dedicated high-temperature calibration data.

### 3.3. Model Training and Results

The 49 calibration samples and 170 simulation samples were combined, giving 219 samples for model training. Three quantities serve as input features: temperature, WOB voltage, and torque voltage; the output targets are the WOB and torque loads. All variables were normalized to zero mean and unit variance, with the normalization statistics computed on the training fold only. The simulation data are finite-element approximations whose voltage scale is inconsistent with the calibration scale, as noted in Section 3.2. Different weights are assigned to the two data sources during training. A weight of 1.0 is assigned to the calibration data, which serve as the directly measured reference, whereas the simulation data are given a weight of 0.6. The influence is verified by the sensitivity analysis below. Each sample is additionally weighted by a temperature-sensitivity factor so that samples farther from the mean temperature receive greater emphasis. A sensitivity analysis over simulation weights from 0 to 2.0 shows that the model ranking and the benefit of hyperparameter optimization are preserved across this range. This result indicates that the conclusions drawn within the calibration domain are robust to the weighting of the simulation data, but the influence of the simulation modeling details on generalization requires a separate analysis, because the simulated voltages differ from the measured voltage scale (Section 3.2); high-temperature calibration data therefore remain necessary for quantitative validation. When excluding the simulation data, the calibration-domain validation RMSE was the lowest and the most stable, confirming that the simulation data were not the source of accuracy in the calibration domain. Their essential role is to provide the only training data carrying temperature variation, which exercises the trend-consistency constraint.

Hyperparameters were selected through nested cross-validation to prevent information leakage: for each of the five outer folds, an inner four-fold cross-validation on the training portion selected the configuration, which comprises the hidden-neuron count L, the regularization coefficient, and the coefficients of the physical constraints, and the selected configuration was then evaluated on the held-out outer fold. All optimizers ran at an equal function-evaluation budget of about 575 evaluations per fold. Seven models were compared under this protocol: a conventional ELM, a PIELM with fixed hyperparameters, and PIELMs optimized by EEFO, IEEFO, and the three single-strategy variants; the mean and standard deviation of the WOB and torque RMSEs across the five outer folds are reported in Table 1.

Table 1 supports three observations. First, hyperparameter optimization is the main source of accuracy: with fixed hyperparameters, the WOB RMSE is 0.712 kN, whereas all optimized variants reduce it to 0.246–0.327 kN, a 2.2–2.9-fold improvement, far below the 3686.8 kN of the conventional ELM, which is ill-conditioned. Second, the optimization outcome is insensitive to the specific strategy: the differences among the three single-strategy variants and the complete IEEFO are all within the fold-to-fold variability. Third, as an additional reference, a ridge-regularized ELM mitigates the ill-conditioning of the conventional ELM through L2 regularization, with its hidden-neuron count and ridge coefficient both selected by inner cross-validation; it reduces the error from 3686.8 kN to 0.708 ± 0.266 kN, showing that ridge regularization is an effective remedy for the ill-conditioned ELM. However, this level is comparable to the 0.712 kN of the fixed-hyperparameter PIELM and far above the 0.246 kN attained after hyperparameter optimization, indicating that classical statistical tuning alone does not reach this accuracy level—within the framework, the physical constraints contribute interpretability while the accuracy gain comes from the hyperparameter optimization of the PIELM. In addition, 25 independent runs on the target problem show that the final fitness values of the five algorithms are very close, within about 2%; that the mean value of IEEFO is the lowest among the five algorithms; and that EEFO and IEEFO do not differ significantly in a two-sided Wilcoxon test, p = 0.252.

**Figure 5:**
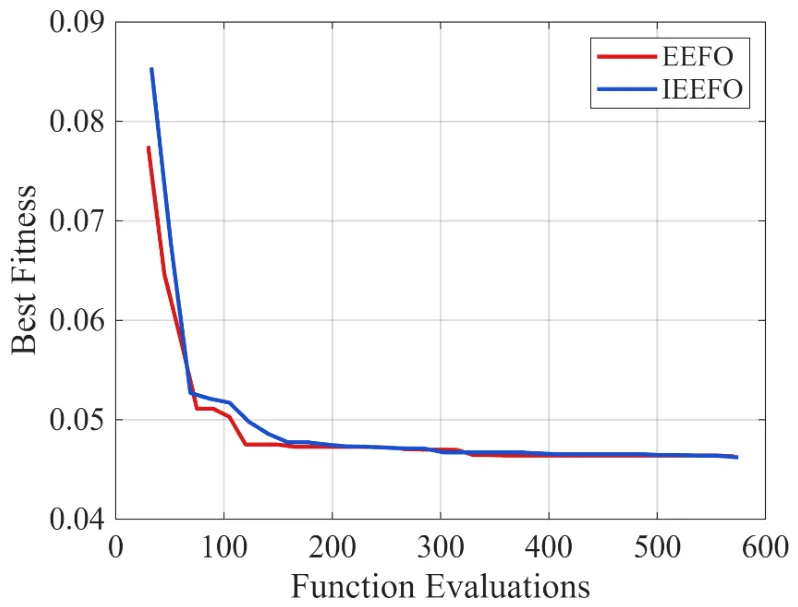
Fitness convergence curves under equal function-evaluation budgets (mean of five cross-validation folds).

Average fitness convergence curves for IEEFO and the standard EEFO under an equal function-evaluation budget are plotted in Figure 5 (mean of the five cross-validation folds; the horizontal axis is the function-evaluation count). The two curves terminate at 575 and 570 evaluations, respectively, so the budgets are aligned; under this equal-budget protocol, the fitness of IEEFO is statistically indistinguishable from that of EEFO (Table 1).

**Figure 6:**
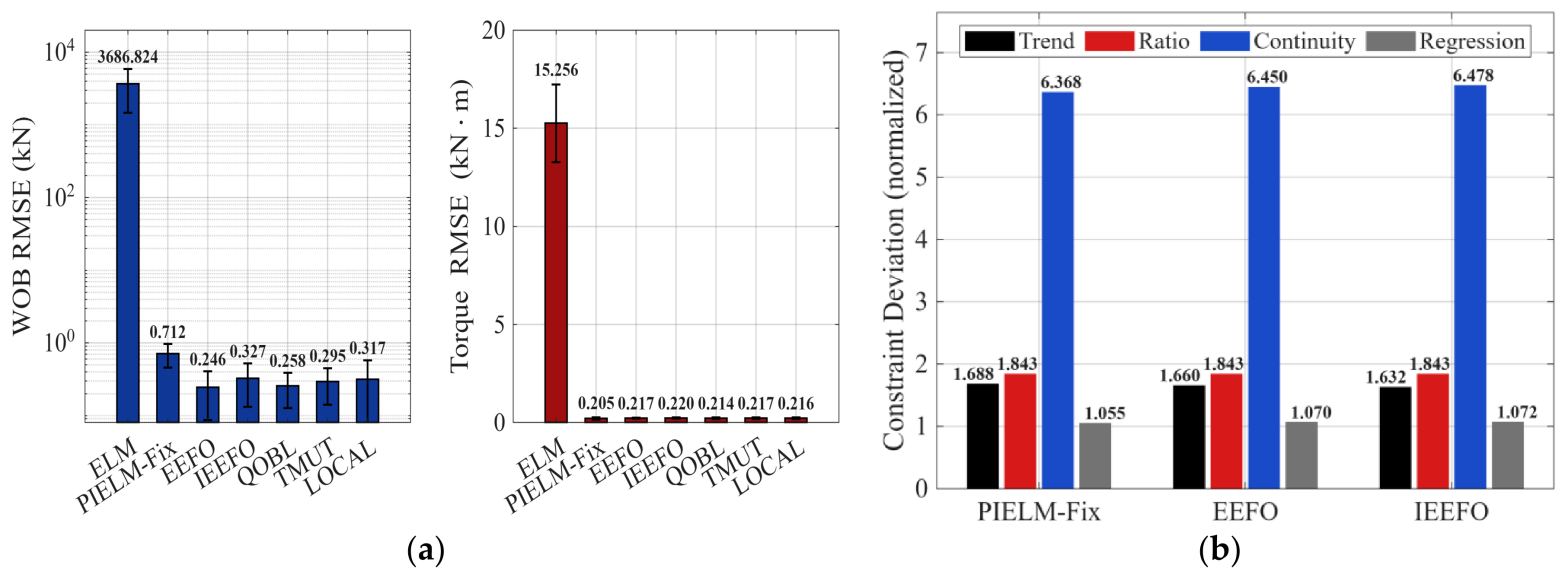
Leakage-free nested cross-validation results: (**a**) WOB and torque RMSE (mean ± standard deviation over five outer folds); (**b**) physical constraint deviations.

Figure 6 summarizes the accuracy distributions and physical-constraint deviations of the models under leakage-free nested cross-validation. Figure 6a shows that the optimized models attain clearly lower WOB RMSE than the PIELM with fixed hyperparameters, which demonstrates the effect of hyperparameter optimization on WOB prediction; the differences between IEEFO and EEFO lie within the fold-to-fold variation. For the torque channel, the differences among the three models are small, indicating that the temperature drift effect of the torque channel is less significant than that of the WOB channel. Figure 6b reports four constraint deviation measures: the mean absolute residual of the embedded trend rows on the training fold, the mean absolute deviation of the predicted voltage ratio from its historical mean in units of the historical standard deviation, the mean squared relative excess of the voltage change rate above its 95th-percentile threshold, and the mean squared normalized deviation from the historical temperature–voltage regression. All four are nonnegative deviations that are well defined on every fold and can vary across models, so the comparison is informative in both directions. A one-sided violation metric of the form max(0, −z) cannot produce a nonzero value when no violation occurs and may even be undefined: for the trend constraint in particular, the temperature is constant at 25 °C across the calibration folds, so any metric built on the temperature–voltage correlation has zero variance and carries no information there. The trend consistency constraint is therefore exercised during training through the simulation samples, the only training data with temperature variation, and is evaluated on the downhole data in the following section.

**Figure 7:**
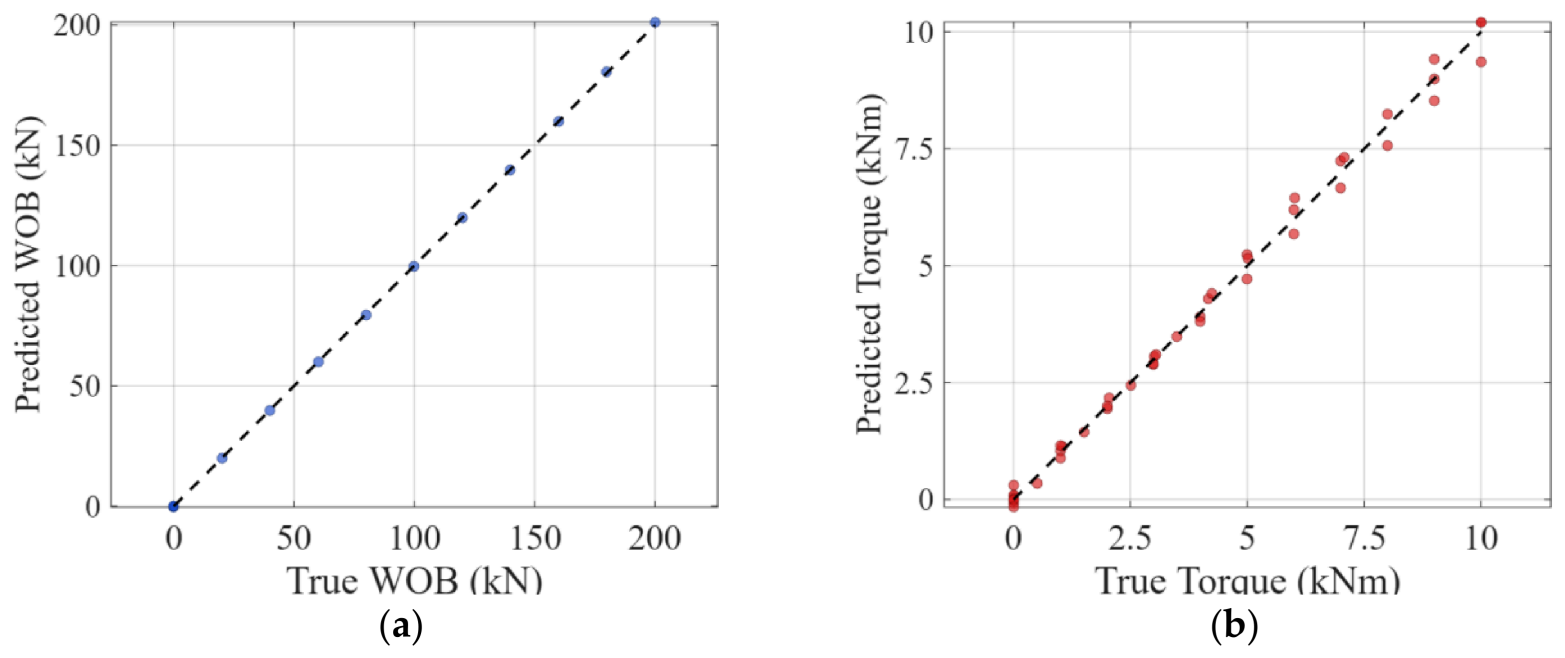
Actual–predicted scatter plots: (**a**) scatter plot of WOB; (**b**) scatter plot of torque.

Figure 7 presents the scatter plots of predicted against ground-truth values across the five validation folds. The dots for WOB and torque lie close to the diagonal line, and no systematic offsets or nonlinear distortions are visible, which shows that the physics-informed extreme learning machine aligns with the calibration data. The WOB channel covers a wider numerical span than the torque channel, which is consistent with the different force levels and measurement limits applied during the calibration experiments.

**Figure 8:**
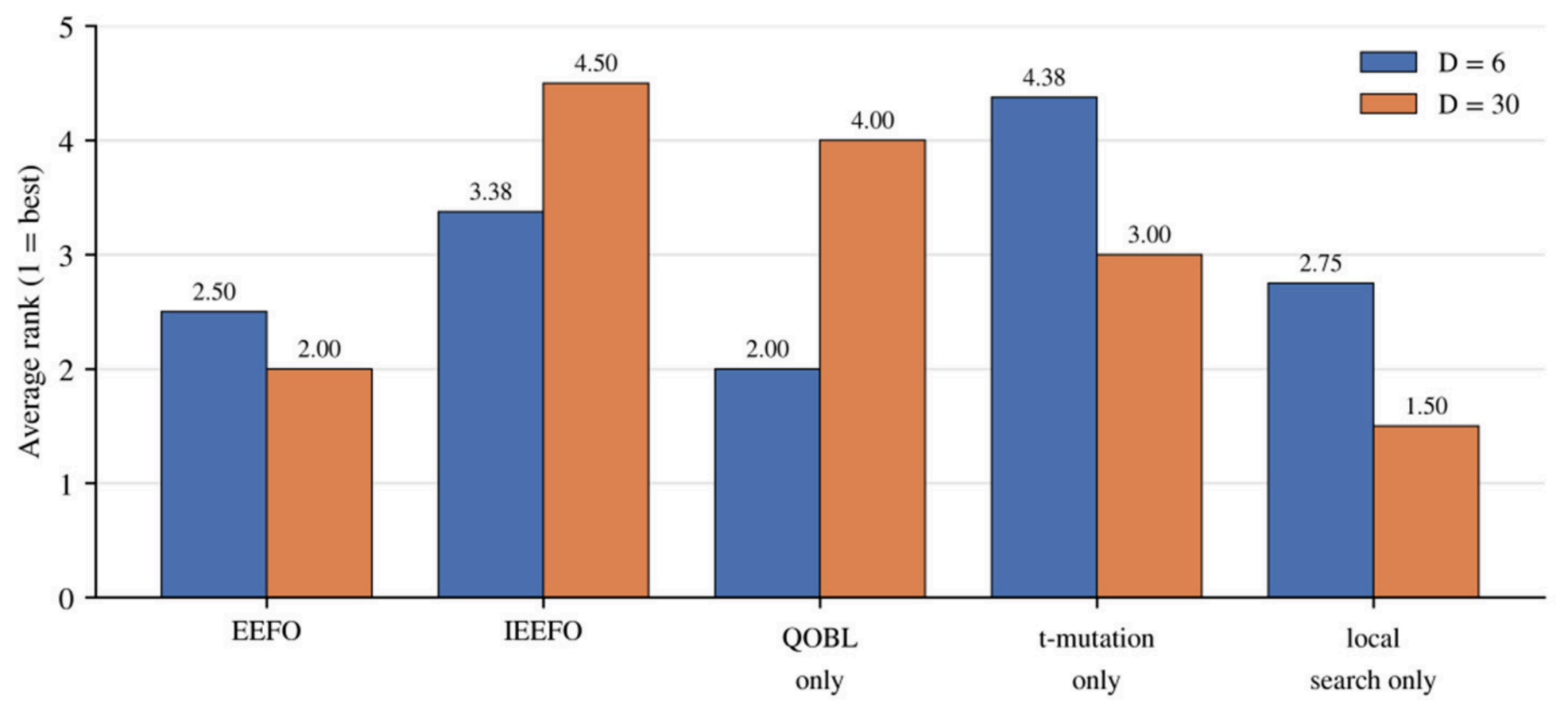
Equal-budget average ranks on the classical benchmark functions (25 independent runs; two-sided Wilcoxon rank-sum test, 0.05 level; 1 = best). At D = 30, the ranks are based on the only two non-saturated functions.

To examine whether the enhancement strategies improve the base algorithm in general, the five algorithms were compared on the 13 classical benchmark functions F1–F13, with average ranks reported in Figure 8. At problem dimension D = 6, the evaluation budget equals that of the target problem, with FE of approximately 575 with a population size of 15; at D = 30, FE is approximately 10,000 with a population size of 30. Each function was run 25 times independently, and two-sided Wilcoxon rank-sum tests were applied at the 0.05 significance level; functions on which all algorithms converge to machine precision were classified as saturated and excluded from ranking. At D = 6, the dynamic-boundary QOBL variant attains the best average rank among the variants at 2.000 on the non-saturated functions, whereas the complete IEEFO attains 3.375; IEEFO is significantly better than EEFO on the Rosenbrock function but significantly worse on four functions: Schwefel 2.22, Schwefel 2.21, Ackley, and Penalized 2. The adaptive t-distribution mutation alone is the weakest variant at 4.375 and is significantly worse than EEFO on four functions—an ablation outcome that supports its exclusion from the final algorithm. At D = 30, 11 of the 13 functions saturate, and on the 2 remaining functions, IEEFO again does not significantly outperform EEFO and is significantly worse on Rosenbrock. Taken together, under an equal function-evaluation budget, none of the enhancement strategies establishes a significant advantage over standard EEFO, and the apparent advantage at equal iteration counts is attributable to the unequal evaluation budgets. At the same time, the ablation provides a clear basis for the configuration choice: the dynamic-boundary QOBL attains the best average rank among the variants at D = 6; the t-distribution mutation alone is significantly worse than standard EEFO; and the final configuration determined by the ablation attains the lowest mean fitness among the five configurations on the target problem, 0.0446 versus 0.0450 for standard EEFO, with a two-sided Wilcoxon test p = 0.252, and it significantly outperforms the initial three-component version on the benchmarks, with three significant wins and no significant loss on the non-saturated functions at D = 6. The deployed model therefore adopts this configuration as the hyperparameter search engine, interchangeable with standard EEFO.

## 4. Measured Data Validation

### 4.1. Preprocessing of Downhole Measurement Data

The sensor systems in the drilling tool collected the downhole data while the drill bit penetrated the deep rock layers, giving an initial record of over one hundred thousand measurements, from which three data streams were extracted: temperature, WOB voltage, and torque voltage. There are no ground-truth load values in the downhole environment; the reference values are therefore calculated from the linear voltage-load calibration relationship obtained in the ground calibration experiments. The downhole environment is characterized by high temperature, severe vibration, and electromagnetic interference, causing the raw signals to contain impulsive spikes and high-frequency fluctuations. The raw signals therefore had to be cleaned before they could be used to evaluate the model. The preprocessing consists of four stages:

1. Rationality Screening. Based on the range of the sensor and the physical limits of the well, records that are obviously unreasonable are eliminated. Samples that fall outside these limits are identified as sensor anomalies or transmission errors and are removed directly. It is a process that eliminates inaccurate entries, which occur when a sensor loses its connection, a cable experiences a short circuit, or the system loses data during transfer. Removing them prevents extreme outliers from being mistaken for normal fluctuations in later processing.
2. Spike Outlier Removal. The 95th percentile of the first-order difference in the voltage signal was used to characterize the normal range of the signal. Five times this value was taken as the spike threshold. When the first-order difference in either channel exceeds the threshold, the current sample and its adjacent samples will be removed together. The value of 5 was verified by sensitivity analysis. This procedure removes only 18 of the 114,985 valid samples, and the results are the same when the multiplier is set to 5, 8, 12, or infinity.
3. Median Filtering. After spike removal, the voltage signal is filtered with a median filter of window size 5 to suppress the high-frequency random noise introduced by the sensor acquisition chain. Median filtering preserves step features more effectively than mean filtering while smoothing the noise. A window of 5 is an empirical balance between smoothing effect and retention of small features. The sensitivity analysis shows that changing the window from 1 to 15 alters the compensated RMSE by less than 0.1%.
4. Window-Based Downsampling. The preprocessed data still retain a high temporal resolution. Inputting the data point by point would be computationally expensive, and more importantly, the short-term operational fluctuations would mask the macroscopic trend of the temperature drift. Every 20 consecutive original sampling points were therefore compressed into one representative point, and the window mean of each channel was taken as the downsampled feature. The block length is the most influential preprocessing parameter, but changing it from 1 to 100 points alters the compensated RMSE by at most 2% and does not change any qualitative conclusion.

### 4.2. Temperature Drift Compensation Results

This section applies the calibration-domain compensation model to the measured downhole data and examines two aspects: the robustness of the error–temperature relationship under different mapping protocols, and the satisfaction of the physical constraints. No ground-truth load measurements are available in the downhole records, and the reference values must therefore be derived from the voltage signals. For the torque channel, the reference is computed from a linear function of the torque voltage, so they are deterministically related (residual about 1 × 10^−14^), and its RMSE is reported only for completeness. The downhole validation focuses on consistency rather than independent accuracy assessment.

Evaluating the predictions requires an affine mapping between the PIELM outputs and the reference values, and the data on which this mapping is fitted strongly affect the result. Because the same samples must not be used both to fit the mapping and to evaluate it, four mapping protocols are compared: P1, with the linear-calibration baseline and the affine prediction-reference mapping both fitted on the evaluation set itself (in-sample mapping); P2, with the mapping fitted on the coldest 10% of the downhole data and applied to the remaining 90%; P3, with the mapping fitted on the first 10% of the time series and applied to the remaining 90%; and P4, with the mapping fitted on the calibration set only, so that no downhole information enters the evaluation.

**Figure 9:**
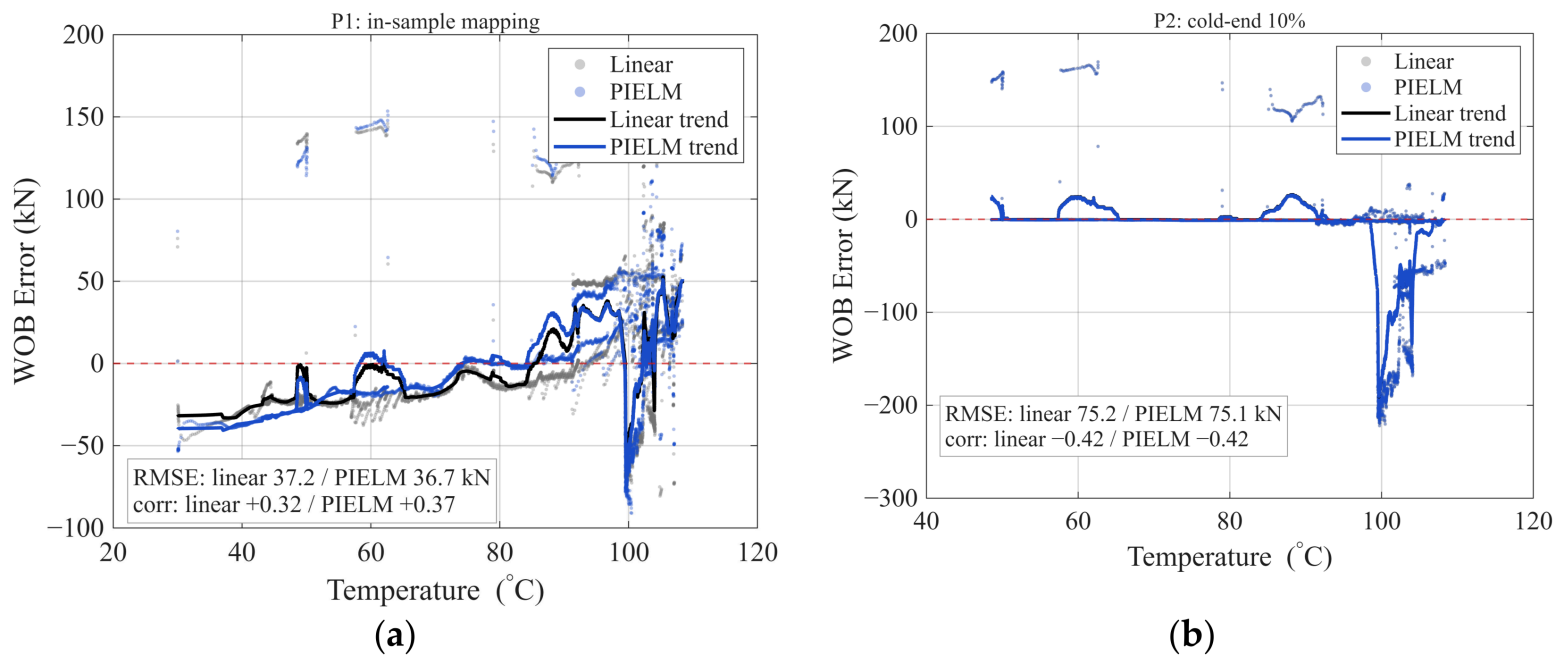
WOB error–temperature distributions: (**a**) P1, in-sample mapping; (**b**) P2, coldest 10% fit.

Figure 9 shows the comparison of the WOB error-temperature scatter and moving-average curves under two mapping protocols; the linear-calibration baseline is taken directly from the voltage-load relationship of the ground calibration experiment, and its error still contains clear temperature-related drift. Figure 9a shows the in-sample mapping (P1): after compensation, the error points concentrate toward zero, and the moving-average curve becomes flatter. Figure 9b shows the coldest 10% mapping (P2): once the mapping does not draw on the evaluated samples, this apparent improvement weakens markedly, in line with the 0.3% apparent elimination reported in Table 2.

Table 2 gives the apparent temperature-drift elimination rate for the four protocols. The rate is defined as the relative decrease in the absolute error–temperature correlation from before to after compensation, with the pre-compensation value as the baseline: a positive value means the correlation weakens, and a negative value means it strengthens. The result depends strongly on the mapping protocol. For P1, the WOB elimination rate is −17.9%: the correlation rises from 0.315 to 0.372, so the apparent drift increases. The P1-fitted slope is −0.0465; its reciprocal corresponds to a 21.5-fold rescaling of the predictions with a sign reversal, so this negative value reflects the flexibility of the in-sample mapping rather than a real change in the error. Under the leakage-free protocols P2–P4, the WOB rates are 0.3%, 0.0%, and 5.1%, all close to zero; under P3, the correlations before and after compensation are both −0.416. The P4 mapping is essentially the identity, with a slope of 1.0001, which shows that the model output is already on the correct load scale in the calibration domain. The torque column shows the same protocol sensitivity, with values of −105.7%, 8.8%, 7.9%, and −32.1%; its reference is determined linearly from the torque voltage and is not an independent measurement, so the column is reported for completeness only.

Table 3 gives the coverage of the downhole inputs by the training domain. The training-domain range is the union of the calibration and simulation samples on each channel, and the in-domain fraction is the share of downhole samples that fall inside it. The downhole temperature range, 29.8–108.6 °C, lies entirely inside the training range of 25.0–120.0 °C, with 100% coverage, so no extrapolation is needed in temperature. For the torque voltage, 99.99% lies inside the training domain and only 0.01% exceeds the training maximum. The WOB voltage is different: its downhole range, 124.5–441.1 mV, lies entirely below the training range of 1189.1–1436.2 mV, and its maximum is only 37% of the training lower bound, so this channel is a full extrapolation. The training data are also confounded: the calibration set sits at a constant 25 °C while the simulation set covers 50–120 °C, and the two occupy disjoint voltage intervals, so temperature and voltage scale are entangled in training. This is why the downhole WOB claim is limited to a consistency check rather than an accuracy assessment.

The apparent elimination rate is reported only as a descriptive diagnostic and is not used to support quantitative claims about compensation accuracy.

**Figure 10:**
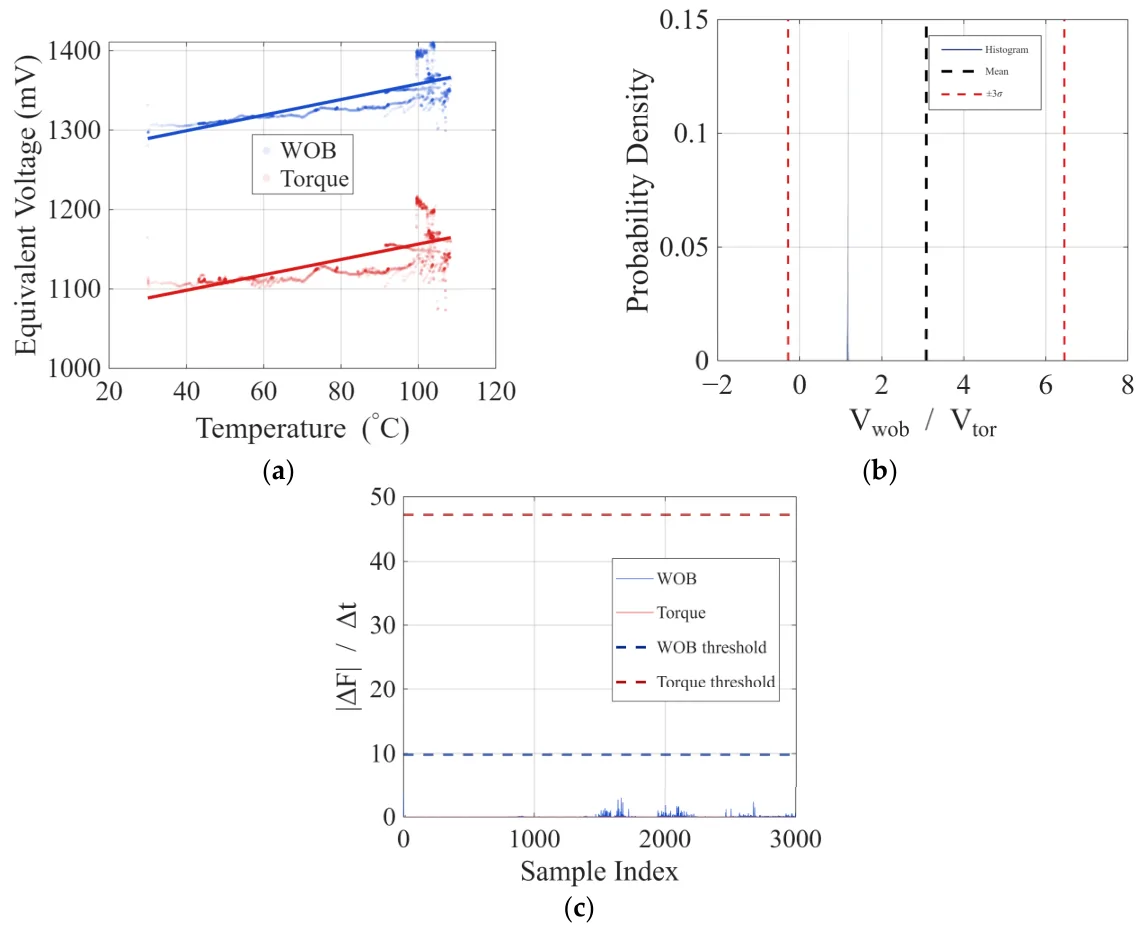
Verification results of three physical constraints: (**a**) prediction scatter plots and fitting trend lines; (**b**) histogram distribution of predicted voltage ratios; (**c**) absolute first-order difference sequences of predicted values.

The trend, ratio, and continuity constraints correspond to the three basic requirements of the downhole process, and Figure 10 gives the verification results.

Figure 10a shows the equivalent voltage of WOB and torque against temperature, with linear fits superimposed, to check the trend-consistency constraint. The reference sign is taken from the original voltage–temperature correlation. When the fitted slope has the same sign as this reference, the model has not introduced a non-physical prediction bias. For both channels, the direction of the temperature–voltage correlation agrees with the downhole reference, so the model keeps the physical fidelity of the signal while compensating.

Figure 10b is the histogram of the PIELM voltage ratio, with the downhole mean and the ±3σ limits superimposed, to check the proportional relationship. During drilling, WOB and torque are coupled through the shared load path, and the voltage ratio fluctuates around its mean under normal conditions. The predicted ratio is well concentrated and almost all values fall inside the limits, which indicates that the joint WOB–torque output agrees with the downhole coupling and shows no alternating imbalance between the two channels.

Figure 10c plots the absolute first-order difference in the predicted WOB and torque values; the dashed line is the 95th-percentile change-rate threshold of the downhole data, used to check continuity. Mechanical inertia limits the instantaneous change rate of the downhole force signals. If the compensation introduced high-frequency fluctuations or step jumps, the absolute difference would grow abnormally and break the temporal smoothness. Only a small fraction of the values exceed the threshold in both channels, so the compensation does not add obvious abnormal fluctuations to the original signal.

## 5. Conclusions

To address temperature drift in bit parameter recorders during deep-well drilling in high-temperature environments, this paper proposes a temperature drift compensation method combining EEFO and PIELM. Physical information related to sensor temperature drift was introduced into the model training through weak constraints, and EEFO was used to optimize the PIELM hyperparameters automatically. The method was evaluated using calibration experiments, thermo-mechanical simulation data, and measured downhole data. The main conclusions are as follows:

1. Four weak physical constraints, namely trend consistency, regression deviation, proportional relationship, and physical process continuity, were incorporated into the PIELM training process in two forms: the trend consistency and regression deviation constraints were embedded into the output-weight solution through an augmented linear system, whereas the proportional relationship and continuity constraints were added to the fitness function as penalty terms, forming the PIELM-based temperature drift compensation model. Calibration and simulation samples were used together in training. PIELM fitted both WOB and torque channels well, and the WOB channel showed a larger RMSE drop, consistent with its stronger drift. Under leakage-free nested cross-validation, the RMSE of WOB is 0.246 ± 0.160 kN for the EEFO-optimized model, while it is 0.712 ± 0.254 kN for the fixed-hyperparameter PIELM, which is a 2.9-fold improvement. The ridge-regularized ELM baseline attains 0.708 ± 0.266 kN, indicating that the accuracy gain stems from the hyperparameter optimization itself, with the physical constraints contributing interpretability on the calibration domain.
2. IEEFO is developed by combining dynamic-boundary quasi-opposition-based learning with an adaptive local fine-search strategy, and the deployed configuration is determined by an equal-budget ablation: the dynamic-boundary QOBL variant attains the best average rank among the variants, and the t-distribution mutation is excluded because it is significantly worse than standard EEFO. The final configuration achieved the lowest mean fitness among the five configurations for the target problem (not significantly different from standard EEFO, *p* = 0.252) and was significantly superior to the initial three-component version on the benchmarks. The verification of 25 independent runs confirmed that the 2.9-fold accuracy gain originates from the hyperparameter optimization itself.
3. A consistency-verification framework was adopted for the downhole verification. The protocol study of the affine mapping indicates that the apparent drift-elimination rate is an artifact of in-sample fitting. The elimination rates under leakage-free mapping protocols are 0.3%, 0.0%, and 5.1%. Moreover, the calibration mapping is essentially the identity mapping, indicating that the downhole WOB predictions are pure extrapolation, as the downhole WOB voltage range lies entirely outside the training range. The physical-constraint checks remain valid: the compensated outputs keep the historical trend directions, ratio distribution, and temporal continuity, which demonstrates the interpretability of the framework.

This study provides an interpretable and practical solution for the compensation of temperature drift in deep-well bit parameters. The method nonetheless has well-defined boundaries. The construction of physical constraints relies on the prior understanding of the temperature drift mechanism; when the sensor structure or installation changes, the constraint forms must be reconstructed. In addition, the downhole evaluation is restricted to consistency checks because no load ground truth is available, and the simulation-based training data carry little temperature-drift information (temperature main effect of 0.00–0.41%). The predictive performance demonstrated in this study is therefore confined to the calibration domain; the performance under actual high-temperature downhole conditions requires dedicated high-temperature validation before it can be quantified, and quantitative accuracy claims are restricted accordingly. Extending the validated domain to the full downhole temperature range requires dedicated high-temperature calibration and field data, which this study identifies as the primary direction for future work. The planned work will also cover online incremental learning and richer physical constraint forms, such as thermo-mechanical coupled partial differential equation residuals, to improve the model’s performance and adaptability in complex deep-well drilling environments.

## Figures and Tables

**Table 1:** Leakage-free nested cross-validation results. RMSE values are reported as mean ± standard deviation across folds; L* and FE are the fold-averaged hidden-neuron count and function-evaluation count.

Model	WOB RMSE (kN)	Torque RMSE (kN·m)	L*	FE
Conventional ELM	3686.8 ± 2218.3	15.26 ± 1.99	6.6	209
PIELM, fixed hyperparameters	0.712 ± 0.254	0.205 ± 0.061	80	1
PIELM + EEFO	0.246 ± 0.160	0.217 ± 0.028	42.2	570
PIELM + IEEFO	0.327 ± 0.194	0.220 ± 0.033	71.2	575
PIELM + QOBL only	0.258 ± 0.130	0.214 ± 0.034	103.6	573
PIELM + t-distribution mutation only	0.295 ± 0.154	0.217 ± 0.032	101.6	570
PIELM + local search only	0.317 ± 0.260	0.216 ± 0.037	71.2	575

**Table 2:** Apparent temperature-drift elimination rate under the four mapping protocols.

Protocol	Apparent WOB Elimination (%)	Apparent Torque Elimination (%)
P1: in-sample mapping	–17.9	–105.7
P2: cold-end 10%	0.3	8.8
P3: time-front 10%	0.0	7.9
P4: calibration-only	5.1	–32.1

**Table 3:** Coverage of the downhole inputs by the training domain (calibration and simulation samples).

Channel	Training Range	Downhole Range	In-Domain Fraction
Temperature (°C)	25.0–120.0	29.8–108.6	100.00%
WOB voltage (mV)	1189.1–1436.2	124.5–441.1	0.00%
Torque voltage (mV)	304.8–1575.1	789.4–2057.5	99.99%

## Data Availability

The original contributions presented in this study are included in the article. Further inquiries can be directed to the corresponding author.
